# Effect of Selected Organic Solvents on Hydroxyl Radical-Dependent Light Emission in the Fe^2+^-EGTA-H_2_O_2_ System

**DOI:** 10.3390/molecules29235635

**Published:** 2024-11-28

**Authors:** Krzysztof Sasak, Michał Nowak, Anna Wlodarczyk, Agata Sarniak, Dariusz Nowak

**Affiliations:** 1 Department of Medical Imaging Techniques, Medical University of Lodz, Lindleya 6, 90-131 Lodz, Poland; krzysztof.sasak@umed.lodz.pl; 2 Radiation Protection, University Hospital No 2, Medical University of Lodz, Zeromskiego 113, 90-549 Lodz, Poland; m.nowak@skwam.lodz.pl; 3 Department of Sleep Medicine and Metabolic Disorders, Medical University of Lodz, Mazowiecka 6/8, 92-215 Lodz, Poland; anna.wlodarczyk@umed.lodz.pl; 4 Department of Clinical Physiology, Medical University of Lodz, Mazowiecka 6/8, 92-215 Lodz, Poland; agata.sarniak@umed.lodz.pl

**Keywords:** chemiluminescence, Fenton system, hydroxyl radicals, organic solvents, aprotic solvents

## Abstract

Numerous compounds that are scavengers of hydroxyl radicals (•OH) in Fenton systems have low solubility in water. Therefore, they are dissolved in organic solvents to reach suitable concentrations in the reaction milieu of the Fenton system. However, these solvents may react with •OH and iron, leading to significant errors in the results. We evaluated 11 solvents (4 alcohols, acetone, 4 esters, dimethyl-sulfoxide, and acetonitrile) at concentrations ranging from 0.105 µmol/L to 0.42 µmol/L to assess their effects on light emission, a recognized measure of •OH radical activity, in the Fe^2+^-EGTA-H_2_O_2_ system. Six solvents inhibited and four solvents enhanced light emission at all tested concentrations. Acetonitrile, which initially suppressed light emission, lost this effect at a concentration of 0.105 µmol/L, (−1 ± 13 (2; 0) %, *p* > 0.05). Methanol, at the lowest tested concentration, inhibited light emission by 62 ± 4% (*p* < 0.05), while butyl butyrate enhanced it by 93 ± 16% (*p* < 0.05). These effects may be explained by solvent-driven •OH-scavenging, inhibition or acceleration of Fe2+ regeneration, or photon emission from excited solvent molecules. Our findings suggest that acetonitrile seems suitable for preparing stock solutions to evaluate antioxidant activity in the Fe^2+^-EGTA-H_2_O_2_ system, provided that the final concentration of this solvent in the reaction milieu is kept below 0.105 µmol/L.

## 1. Introduction

Hydroxyl radicals (•OH) are a type of reactive oxygen species (ROS) and are considered the most reactive ROS [1]. The chronic overproduction of ROS in tissues is believed to contribute to various pathologies including persistent inflammation, cancer, autoimmune disorders, as well as cardiovascular and neurological diseases [2,3,4,5,6]. Thus, normalizing ROS levels in the body could have beneficial effects in preventing and treating these diseases [7,8]. One potential approach is an introduction of chemical (or phytochemical) compounds into the body that can react with ROS, forming non-reactive or less reactive derivatives, thereby suppressing tissue ROS levels.

Candidate compounds for such therapies should be tested in vitro with systems generating individual ROS (e.g., •OH, H_2_O_2_, superoxide radical, singlet oxygen, hypochlorite, or peroxynitrite) before proceeding to experiments in cell cultures, animal models, and clinical trials to assess their safety and antioxidant effectiveness. The Fenton system, composed of Fe^2+^ and H_2_O_2_, or modified versions such as the Fe^3+^-EDTA (ethylenediaminetetraacetic acid)-H_2_O_2_-ascorbate system is a simple and effective generator of •OH radicals in aqueous solutions and is commonly used to evaluate the anti-•OH (antioxidant) activity of various compounds [9].

Since the direct detection of •OH radicals in aqueous environments is not feasible [10], numerous indirect methods have been developed to measure OH activity. These methods typically involve adding specific •OH probes (e.g., 2-deoxy-D-ribose, N, N’-(5-nitro-1,3-phenylene)-bis-glutaramide, salicylic acid, coumarin, dimethyl-sulfoxide, phthalhydrazide, and rhodamine-based probes) that react with •OH to form by-products in the reaction milieu. The level of these by-products is measured using techniques like spectroscopy, fluorescence, luminescence, or electron spin resonance (ESR) and is used as an indicator of the •OH activity in a given Fenton system with and without the tested antioxidant [9,10,11,12,13,14].

Plant polyphenols, which are recognized as natural antioxidants [15], have been extensively studied for their ability to protect biomolecules from peroxidative damage and therefore play a significant role in preventive medicine [16,17]. Over 8000 phenolic compounds have been identified in various plant species [16]. Since most polyphenols are highly soluble in organic solvents, they are typically extracted from plant materials using solvents such as methanol, ethanol, ethyl acetate, or acetone [18]. Stock solutions of polyphenols are often prepared in organic solvents for in vitro experiments because these solutions are more stable and convenient for laboratory work.

However, using these stock solutions or their aqueous dilutions introduces organic solvents into the Fenton system along with the tested phenolics, which can be the factor responsible for the apparent augmentation of its antioxidant activity or masking of its pro-oxidant effect. To overcome this problem, additional control systems are needed, including experiments with the solvent alone, the solvent with the Fenton system, and the solvent with the incomplete Fenton system. Furthermore, possible interactions between the solvent and the •OH probe should also be excluded. The common approach of subtracting the results obtained for a sample containing the Fenton system, •OH probe, and solvent from those for a mixture of the Fenton system, •OH probe, solvent, and the tested compound introduces errors because it does not account for the differing rates of reaction between the solvent and the tested compound with •OH radicals.

Recently, we developed a modified Fenton system composed of Fe^2+^-EGTA (ethylene glycol-bis (β-aminoethyl ether)-N, N, N, N -tetraacetic acid) and H_2_O_2_ (Fe^2+^-EGTA-H_2_O_2_ system). In this system, •OH radicals generated through a Fenton reaction can react with ether bonds in an EGTA backbone structure, forming products that contain triplet-excited carbonyl groups [19]. The transition of these carbonyl groups from the excited state to the ground state is accompanied by ultra-weak chemiluminescence (UPE), which serves as an indirect measure of •OH radical activity [19]. By using the Fe^2+^-EGTA-H_2_O_2_ system, we were able to evaluate the pro-oxidant (enhancing UPE) and antioxidant (inhibiting UPE) activities of aqueous solutions of various plant polyphenols [20] and ascorbic acid [21].

We also discovered that dimethyl sulfoxide (DMSO), a commonly used solvent for phenolic solutions, inhibits UPE in the Fe^2+^-EGTA-H_2_O_2_ system [19]. This finding suggests that antioxidant samples containing various solvents may produce results with significant errors. Therefore, this study aimed to evaluate the effect of six typical organic solvents (methanol, ethanol, acetone, ethyl acetate, propan-1-ol, and n-pentanol) and two polar aprotic solvents (acetonitrile and DMSO) at three concentrations (0.105 µmol/L, 0.21 µmol/L, and 0.42 µmol/L) on UPE in the Fe^2+^-EGTA-H_2_O_2_ system. Our goal was to identify solvents that do not affect or minimally affect light emission from the system. Additionally, we tested three less commonly used solvents (benzyl acetate, amyl acetate, and butyl butyrate) to provide further insights into the potential mechanisms behind the effects of these solvents on UPE in the Fe^2+^-EGTA-H_2_O_2_ system.

## 2. Results

All the tested organic solvents demonstrated a significant effect on light emission from the Fe^2+^-EGTA-H_2_O_2_ system. Six of them (methanol, ethanol, propan-1-ol, n-pentanol, DMSO, and benzyl acetate) inhibited UPE at all tested concentrations, whereas four (acetone, ethyl acetate, amyl acetate, and butyl butyrate) enhanced the light emission. Only acetonitrile, at the lowest concentration of 0.105 µmol/L, did not affect the light emission; however, at higher concentrations (0.21 µmol/L and 0.42 µmol/L), it showed an inhibitory effect (Table 1 and Table 2). These results indicate that the application of antioxidants (e.g., polyphenols and water-insoluble vitamins) dissolved in organic solvents may lead to significant errors and create difficulties in the interpretation of results when tested in the modified Fe^2+^-EGTA-H_2_O_2_ Fenton system. Moreover, these findings suggest that there is an advantage of using aqueous solutions of potential antioxidants (even at very low concentrations) in testing antioxidant activity with the Fe^2+^-EGTA-H_2_O_2_ system.

The tested solvents were mixed with EGTA and Fe^2+^, followed by either automatic H_2_O_2_ or H_2_O injection. Total light emission was then measured for 120 s. The results are presented as the mean and standard deviation (and median and interquartile range) of the UPE (ultra-weak photon emission, expressed in Relative Light Units) obtained from 7 independent series of experiments. * indicates significant differences (*p* < 0.05) compared to the corresponding values for the Fe^2+^-EGTA-H_2_O_2_ system with the solvent. The incomplete Fe^2+^-EGTA-H_2_O_2_ system with or without the appropriate solvent was used as a control.

### 2.1. Solvents That Inhibited Light Emission from the Fe^2+^-EGTA-H_2_O_2_ System

Table 2 shows the solvents that suppressed UPE in the Fe^2+^-EGTA-H_2_O_2_ system. DMSO, methanol, ethanol, and propan-1-ol exhibited the strongest inhibitory effects on light emission. At the lowest tested concentration of 0.105 µmol/L, the inhibition ranged from 52 ± 3% for ethanol to 67 ± 2% for DMSO, with no significant increase observed at the concentration four times higher than that of the solvent (ethanol 65 ± 1% and DMSO 70 ± 2%) (Table 2). Benzyl acetate and n-pentanol were approximately half as effective (*p* < 0.05) at suppressing UPE in comparison to the four aforementioned solvents. Additionally, their inhibitory effects remained relatively unchanged across the concentration range of 0.105 µmol/L to 0.42 µmol/L. Acetonitrile at a concentration of 0.42 µmol/L was approximately 3 times less effective (*p* < 0.05) at inhibiting light emission than the strongest inhibitor. However, it was the only solvent that did not affect UPE at the lowest concentration of 0.105 µmol/L (Table 2).

### 2.2. Solvents That Enhanced Light Emission from the Fe^2+^-EGTA-H_2_O_2_ System

Butyl butyrate was the strongest enhancer of photon emission in the Fe^2+^-EGTA-H_2_O_2_ system. At a concentration of 0.42 µmol/L, this solvent increased UPE by approximately fivefold (Table 3). The enhancing effect was concentration-dependent, with UPE increasing by only 1.93 times at the lower concentration of 0.105 µmol/L. The other solvents did not show a clear concentration-dependent effect. However, ethyl acetate exhibited a reduced enhancing effect at 0.21 µmol/L compared to 0.105 µmol/L and 0.42 µmol/L (*p* < 0.05) (Table 3). The remaining two solvents, acetone and amyl acetate, showed a moderate but consistent stimulation of UPE, with the mean enhancement not exceeding 40% at any tested concentration (Table 3).

The tested solvent was mixed with EGTA and Fe^2+^, followed by the automatic injection of H_2_O_2_. The total light emission was then measured for 120 s. The results are presented as the mean and standard deviation (and median and interquartile range) of the percentage inhibition of light emission, which was obtained from seven independent experiments. * indicates significant inhibition (*p* < 0.05) compared to the UPE of the Fe^2+^-EGTA-H_2_O_2_ system without the solvent.

## 3. Discussion

### 3.1. Solvents That Inhibited Light Emission from the Fe^2+^-EGTA-H_2_O_2_ System

Four of the seven solvents that inhibited UPE in the Fe^2+^-EGTA-H_2_O_2_ system are aliphatic alcohols: methanol, ethanol, propan-1-ol, and n-pentanol. Methanol, ethanol, and propane-1-ol exhibited similar inhibitory effects on UPE in the Fe^2+^-EGTA-H_2_O_2_ system, whereas n-pentanol was almost two-fold weaker in inhibiting •OH-dependent light emission across all the concentrations tested. Additionally, the percentage inhibition of UPE remained nearly constant regardless of the concentration for all four alcohols.

The reaction of aliphatic alcohols with •OH radicals involves hydrogen abstraction from both the hydroxyl (-OH) group and the aliphatic carbon chain that lacks the -OH group [22].

R-OH + •OH → R-OH ∙∙∙ •OH (formation of the pre-reactive complex) (R=CH_3_, C_2_H_5_, C_3_H_7_) (1)

R-OH ∙∙∙ •OH → R=O + H_2_O (hydrogen abstraction from -OH group) (2)

R-OH ∙∙∙ •OH → R(-H)-OH + H_2_O (hydrogen abstraction from aliphatic carbon chain that lacks the -OH group) (3)

However, due to the high bond-dissociation energy of the hydroxyl O-H bond, the abstraction of hydrogen from an aliphatic carbon chain that lacks the -OH group by •OH radicals (reaction (3)) is preferred [23]. On the other hand, the formation of acetaldehyde in the course of ethanol oxidation by various systems generating •OH radicals [24] suggests that reaction (2)—hydrogen abstraction from the -OH group—may also occur as a result of the oxidative attack on ethanol (and potentially methanol) in the Fe^2+^-EGTA-H_2_O_2_ system.

**Figure 1:**
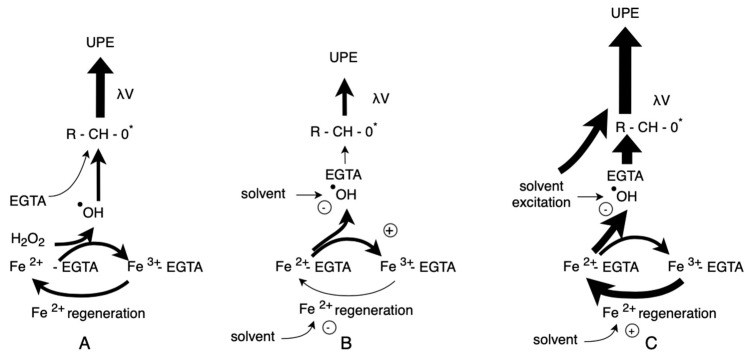
Plausible mechanisms through which organic solvents affect UPE in the Fe^2^⁺-EGTA-H_2_O_2_ System. (**A**) Without the addition of solvent. In this case, the •OH radicals generated by the Fe^2^⁺-EGTA-H_2_O_2_ system attack ether bonds in the EGTA structure leading to the formation of products containing triplet-excited carbonyl groups (R-CH-O*), which results in light emission (UPE—ultra-weak photon emission). Simultaneously, Fe^2^⁺ is regenerated according to the following chemical equations: Fe^3+^-EGTA + O_2_•- → Fe^2+^-EGTA + O_2_ (1); H_2_O_2_ + Fe^3+^-EGTA → HO_2_• + H^+^ + Fe^2+^- EGTA (2); and HO_2_• + Fe^3+^-EGTA → O_2_ + H^+^ + Fe^2+^-EGTA (3). This regenerated Fe^2^⁺ contributes to the further production of •OH radicals and UPE. (**B**) With the addition of an organic solvent that inhibits UPE in the Fe^2^⁺-EGTA-H_2_O_2_ system. The solvent directly scavenges •OH radicals, thereby protecting the ether bonds in EGTA and inhibiting the formation of triplet-excited carbonyl-containing products, which reduces light emission. Some solvents (e.g., ethanol) may also inhibit Fe^2^⁺ regeneration (Equation (1)), further suppressing UPE. (**C**) With the addition of an organic solvent that enhances UPE in the Fe^2^⁺-EGTA-H_2_O_2_ system. The solvent accelerates Fe^2^⁺ regeneration. In some cases, such as with acetone, the solvent itself may emit light due to excitation to a triplet state. However, these solvents can simultaneously scavenge •OH radicals; therefore, the enhancing effect is observed when the rate of •OH radical generation, driven by accelerated Fe^2^⁺ regeneration, exceeds the loss of radicals due to the solvent’s scavenging activity.

Reaction (3) may result in the formation of unsaturated bonds between carbon atoms in the aliphatic chain of alcohols and carbon-centered radicals. These unsaturated bonds could be highly susceptible to further oxidative attack by •OH radicals through addition to the double bond [24]. In methanol, ethanol, and propanol, the rate of hydrogen abstraction by •OH radicals is the highest at the carbon atoms directly attached to the hydroxyl group, whereas, the hydrogen of the -OH group itself is less reactive [24]. The rate constant for the reaction of •OH radicals with alcohols was the highest for methanol, followed by ethanol and propanol [25]. The process of hydrogen abstraction by the •OH radical is a simple atom-transfer reaction in which the bond to the hydrogen atom is broken and a new bond to the oxygen atom of the OH radical is formed [26]. In the case of higher aliphatic alcohols, hydrogen atom abstraction can occur at any carbon atom (the energy barrier for hydrogen abstraction is similar and may result in the formation of various carbon-centered alcohol radicals (e.g., α-radical, β-radical)) [25,26]. This may be explained, for instance, by a similar energy barrier for •OH radical-induced abstraction of Hα (3.487 kcal/mol) and Hβ (8.029 kcal/mol) in ethanol, as well as abstraction of Hα (3.445 kcal/mol), Hβ (6.060 kcal/mol), and Hγ (8.463 kcal/mol) in propanol [26]. Although the bond-dissociation energy of the hydroxyl O-H bond is higher than that of the C-H bond, alkoxyl radicals can be formed in mixtures containing aliphatic alcohols and Fenton systems through the abstraction of hydrogen bonded to the oxygen atoms of the –OH groups [26]. The alcohol radicals can react with each other to give non-radical products [25]. Alcohols (e.g., methanol, ethanol, and propanol) can also react with the hydrogen generated during the regeneration of Fe^2+^ (Figure 1); however, the rate constants for this process are distinctly lower than those between •OH radicals and alcohols [25,27]. The formation of unsaturated bonds in the aliphatic chain of alcohols that are susceptible to oxidative attack by •OH radicals, the comparable bond-dissociation energy of C-H bonds, and the low reactivity of hydrogens in the -OH group may explain the similar inhibitory effects of methanol, ethanol, and propan-1-ol on UPE in the Fe²⁺-EGTA-H₂O₂ system across the tested concentration range of 0.105 µmol/L to 0.42 µmol/L.

In contrast, the higher molecular weight of n-pentanol and thus its lower molecular mobility and difficult access to the Fe^2+^-EGTA complex may result in a weaker inhibitory effect on light emission in the Fe^2+^-EGTA-H_2_O_2_ system.

Taking all the above into consideration, the studied aliphatic alcohols are recognized as effective scavengers of •OH radicals [28], which aligns with the results of our experiments. Ethanol has also been reported to strongly inhibit the reaction of Fe^3+^ with H_2_O_2_ [29], potentially leading to the regeneration of Fe^2+^ and the subsequent generation of •OH radicals as indicated by the following equations [30]:

(4) $$H_{2}O_{2}\;+\;{\mathrm{Fe}}^{3+}-\mathrm{EGTA}\;\overset{\begin{array}{c} ethanol\\ ↓⊖ \end{array}}{\to }\;{HO}_{2}•\;+\;H^{+}\;+\;{\mathrm{Fe}}^{2+}-\mathrm{EGTA}$$

HO_2_^•^ + Fe^3+^-EGTA → O_2_ + H^+^ + Fe^2+^-EGTA (5)

The inhibition of reaction (4) by ethanol, which may be due to the plausible formation of iron in a +5 oxidation state (FeO^3+^) [29], also results in the suppression of reaction (5). Both these processes may additionally contribute to the strong inhibition of UPE in the Fe^2+^-EGTA-H_2_O_2_ system. It cannot also be ruled out that other aliphatic alcohols (e.g., methanol, propan-1-ol, and n-pentanol) may exhibit a similar inhibitory effect on Fe^2+^ regeneration, thereby suppressing light emission in our modified Fenton system. However, further studies are required to confirm this additional inhibitory mechanism.

DMSO is well established as an excellent scavenger of •OH radicals [31] and the reaction between these two molecules leads to the formation of methanesulfinic acid [32,33]. Due to its small molecular size, DMSO can easily access the vicinity of the Fe^2+^- EGTA complex and compete with ether bonds for the •OH radicals, thereby inhibiting UPE in the Fe^2+^-EGTA-H_2_O_2_ system.

Benzyl acetate, an acetate ester of benzyl alcohol, moderately inhibited photon emission from the Fe^2+^-EGTA-H_2_O_2_ system at all the concentrations tested. This suppression is likely due to •OH addition to the aromatic ring and subsequent cleavage of the benzene structure [34,35]. Although acetonitrile can react rapidly with •OH [36,37], little is known about the products of this reaction. It is assumed that the initial attack of •OH radicals on acetonitrile generates similar proportions of CH_2_CN and CH_3_C(OH)N [37]. On the other hand, some data suggest that in the presence of O_2_, these products may secondarily facilitate the formation of •OH radicals [37]. Nevertheless, the inhibitory effect of acetonitrile on the UPE in the Fe^2+^-EGTA-H_2_O_2_ system decreased at lower concentrations and at the concentration of 0.105 µmol/L, no effect on light emission was observed. This indicates that antioxidant solutions in acetonitrile could be suitable for evaluating anti-•OH radicals activity, provided that the final concentration of acetonitrile in the reaction milieu does not exceed 0.105 µmol/L.

### 3.2. Solvents That Enhanced Light Emission from Fe^2+^-EGTA-H_2_O_2_ System

Acetone, ethyl acetate, amyl acetate, and butyl butyrate can all react with •OH radicals [38,39,40,41]. For instance, the reaction of acetone with •OH radicals primarily generates acetonyl radicals and, to a lesser extent, acetic acid (less than 1%) [42]. Therefore, one may expect that acetone and the other three esters would inhibit ultra-weak photon emission (UPE) in the Fe^2+^-EGTA-H_2_O_2_ system. Surprisingly, we observed the opposite effect: these solvents significantly enhanced light emission.

The bidirectional action of these compounds—which involves both scavenging •OH radicals and simultaneously enhancing their generation in the Fe²⁺-EGTA-H₂O₂ system—may explain the observed augmentation of UPE by the aforementioned solvents.

One likely mechanism responsible for this phenomenon is the acceleration of Fe²⁺ regeneration, allowing it to re-enter reactions that lead to the production of •OH radicals, and oxidative attack on the ether bonds in the EGTA backbone structure, resulting in the formation of products containing triplet-excited carbonyl groups and subsequent light emission [19]. This mechanism seems very plausible, particularly because our system had a 28-fold excess of H₂O₂ compared to Fe²⁺ ions. Consequently, each process of reducing Fe³⁺ to Fe²⁺ would enhance the emitted photon signal.

Acetone in combination with urea has been reported to reduce Fe^3+^ to Fe^2+^ under solvothermal conditions [43]. Moreover, acetone alone, as well as ethyl acetate in solutions exposed to light, has also been shown to reduce Fe^3+^ to Fe^2+^ [44,45]. While there is currently no data on the reduction of Fe³⁺ by amyl acetate or butyl butyrate, it cannot be ruled out that these esters may accelerate Fe^2+^ regeneration in the reaction mixture of the Fe^2+^- EGTA-H_2_O_2_ system.

Oxidation processes initiated by oxidants generated in the Fe^2+^-EGTA-H_2_O_2_ system (such as H_2_O_2_ and •OH radicals) could result in the formation of excited products derived from the tested solvents, potentially emitting light and enhancing UPE under the conditions of our experiments. This second mechanism of UPE augmentation appears to be the most likely physicochemical pathway for acetone that may be activated to the triplet state, leading to photon emission [46].

## 4. Material and Methods

### 4.1. Chemicals and Solutions

All chemicals used were of analytical grade. Sodium phosphate monobasic monohydrate (NaH_2_PO_4_ × H_2_O), sodium phosphate dibasic heptahydrate (Na_2_HPO_4_ × 7H_2_O), iron (II) sulfate heptahydrate (FeSO_4_ × 7H_2_O), sodium hydroxide (NaOH), ethylene glycol-bis (β-aminoethyl ether)-N, N, N′, N′-tetraacetic acid (EGTA), methanol, ethanol, acetone, ethyl acetate, propan-1-ol, n-pentanol, acetonitrile, isopentyl acetate, and dimethyl sulfoxide (DMSO) were purchased from Sigma-Aldrich Chemicals (St. Louis, MO, USA). Benzyl acetate and n-butyl butyrate were obtained from Thermo Scientific (Kandel, Germany). A 30% H_2_O_2_ solution (*w*/*w*) was obtained from Chempur (Piekary Slaskie, Poland). Sterile deionized pyrogen-free water (freshly prepared, resistance > 18 MW/cm, HPLC H_2_O Purification System, USF Elga, Buckinghamshire, UK) was used throughout the study. Working aqueous solutions of 22.7 µmol/L, 11.35 µmol/L, and 5.675 µmol/L organic solvent (methanol, ethanol, acetone, ethyl acetate, propane-1-ol, n-pentanol, isopentyl acetate, benzyl acetate, and n-butyl butyrate) and aprotic solvent (acetonitrile, DMSO) were prepared before the assay. Other working solutions (5 mmol/L FeSO_4_,10 mmol/L EGTA, and 28 mmol/L H_2_O_2_) and 10 mmol/L phosphate buffer (pH = 6.6) were prepared as previously described [47].

### 4.2. Effect of Selected Solvents on Light Emission by the Fe^2+^-EGTA-H_2_O_2_ System

Chemical reactions in the 92.6 μmol/L Fe^2+^-185.2 μmol/L EGTA-2.6 mmol/L H_2_O_2_ system, which lead to ultraweak photon emission (UPE), were conducted in 10 mmol/L phosphate buffers (pH = 6.6) with and without the addition of organic solvents. UPE was measured using a multitube luminometer (AutoLumat Plus LB 953, Berthold, Germany), which was equipped with a Peltier-cooled photon counter covering a spectral range from 380 to 630 nm and operating at a temperature of 8 °C to ensure high sensitivity as well as a low and stable background noise levels.

Briefly, 20 μL of a 10 mmol/L EGTA solution was added to a tube (Lumi Vial Tube, 5 mL, 12 × 75 mm, Berthold Technologies, Bad Wildbad, Germany) containing 940 μL of a 10 mmol/L phosphate buffer (pH 6.6). Next, 20 μL of a 5 mmol/L FeSO_4_ solution was added, and after gentle mixing, the tube was placed in the luminometer and incubated for 10 min in the dark at 37 °C. Then, 100 μL of a 28 mmol/L H_2_O_2_ solution was added via an automatic dispenser, and the total light emission (expressed in Relative Light Units—RLU) was measured for 120 s. This measurement represented the baseline signal generated by the complete reaction system in the absence of a solvent. The final concentrations of FeSO_4_, EGTA, and H_2_O_2_ in the reaction mixture were 92.6 µmol/L, 185.2 µmol/L, and 2.6 mmol/L, respectively.

To evaluate the solvent’s effect on the UPE of the Fe^2^⁺-EGTA-H_2_O_2_ system, 20 µL of the working solvent solution was added immediately after the FeSO_4_ solution. The solvent was prepared at three initial concentrations: 22.7 µmol/L, 11.35 µmol/L, and 5.675 µmol/L in water. This resulted in three final solvent concentrations in the reaction mixture: 0.105 µmol/L, 0.21 µmol/L, and 0.42 µmol/L. The controls included an incomplete system (Fe^2^⁺-EGTA-H_2_O, where H_2_O_2_ was not introduced) and an incomplete system with a solvent (Fe^2^⁺-EGTA-solvent- H_2_O, where H_2_O_2_ was also omitted) (Table 2). The number of control experiments was reduced compared to our previous study [21], as these experiments had previously excluded the possible interactions of the tested compounds with H_2_O_2_ alone (which did not produce any spurious light emission) and the contribution of singlet-oxygen decay to UPE in the Fe^2^⁺-EGTA-H_2_O_2_ system [48]. The percentage inhibition and enhancement of UPE by a given solvent were calculated using the following formulas:

% inhibition = (UPE of Fe^2^⁺-EGTA-H_2_O_2_) − (UPE of Fe^2^⁺-EGTA-solvent-H_2_O_2_)/(UPE of Fe^2^⁺-EGTA-H_2_O);

% enhancement = (UPE of Fe^2^⁺-EGTA-solvent-H_2_O_2_) − (UPE of Fe^2^⁺-EGTA-H_2_O_2_)/(UPE of Fe^2^⁺-EGTA-H_2_O).

The results were obtained from 7 series of separate experiments. In one set of experiments, all the samples (1–4; Table 4) were analyzed simultaneously to minimize inter-assay variability.

The working solutions were mixed in the following order: 10 mM/L phosphate buffer (PB, pH 6.6); 10 mmol/L aqueous solution of ethylene glycol-bis(β-aminoethyl ether)-N, N, N′, N′-tetraacetic acid (EGTA); 5 mmol/L solution of FeSO_4_; and 22.7 µmol/L, 11.35 µmol/L, or 5.675 µmol/L solvent dissolved in water. The final concentrations of the solvent in the entire reaction mixture (1080 µL) were 0.105 µmol/L, 0.21 µmol/L, and 0.42 µmol/L. After gentle mixing, the tube was placed into the luminometer, incubated for 10 min at 37 °C in the dark, and then (28 mmol/L aqueous solution of H_2_O_2_) or (H_2_O) was automatically injected using a dispenser, and the total light emission was measured for 2 min.

### 4.3. Statistical Analyses

The results (UPE—total light emission) for the % inhibition or % enhancement by the solvent are expressed as relative light units (RLU) and shown as the means (and standard deviations) and medians and interquartile ranges (IQRs). Comparisons between the UPE of the Fe^2+^-EGTA-H_2_O_2_ system and the light emission from the corresponding samples of the modified system (a complete system with solvent or incomplete system with and without solvent) were analyzed using the independent-sample (unpaired) *t*-test or the Mann–Whitney U test, depending on the data distribution, which was tested using the Kolmogorov–Smirnov–Lilliefors test. The Brown–Forsythe test, used to analyze the equality of the group variances, was applied before the unpaired *t*-test. If the variances were unequal, Welch’s *t*-test was used instead of the standard *t*-test. Comparisons of the % inhibition or % enhancement of UPE caused by the evaluated organic solvents were analyzed in the same way. A *p*-value < 0.05 was considered significant.

## 5. Conclusions

All the tested solvents (n = 11) affected the UPE of the Fe^2+^-EGTA-H_2_O_2_ system. Seven of the solvents inhibited photon emission from the modified Fenton system, while four enhanced it. These effects were observed at all three tested concentrations (0.105 µmol/L, 0.21 µmol/L and 0.42 µmol/L). Notably, only acetonitrile at the lowest concentration of 0.105 µmol/L completely lost its inhibitory effect on UPE in the Fe^2+^-EGTA-H_2_O_2_ system. This suggests that stock solutions of the tested compounds in acetonitrile could be used under conditions where the final concentration of this solvent in the reaction milieu is below 0.105 µmol/L.

Figure 1 summarizes the inhibitory (Figure 1B) and enhancing (Figure 1C) effects of the tested solvents on UPE. Inhibition was primarily attributed to the direct scavenging of •OH radicals, while the enhancement of photon emission appeared to be due to accelerated regeneration of Fe^2^⁺ ions.

In a recent study, we examined the •OH-scavenging properties of various polyphenols using the Fe^2^⁺-EGTA-H_2_O_2_ system. Aqueous polyphenol solutions (without organic solvents) exhibited both pro- and antioxidant activities across a concentration range of 5–50 µmol/L [20]. Five of the seventeen tested polyphenols at a concentration of 5 µmol/L showed no significant effect on UPE in the Fe^2+^-EGTA-H_2_O_2_ system [20]. In contrast, 10 of the 11 tested organic solvents significantly modulated (either inhibited or enhanced) the •OH-dependent UPE at much lower concentrations (0.105 µmol/L)—almost 47 times lower than the polyphenol concentrations. This finding suggests that organic solvents are more potent modulators of •OH-induced reactions in the Fe^2+^-EGTA-H_2_O_2_ system than some phytochemicals (e.g., polyphenols and vitamins) [19,21].

Furthermore, it can be concluded that the most suitable approach for evaluating the pro- or antioxidant activities of various compounds in the Fe^2^⁺-EGTA-H_2_O_2_ system, or in other modified Fenton systems, is to use aqueous solutions of these compounds. While aqueous polyphenol solutions may result in lower compound concentrations in the in vitro reaction mixture compared to organic solvent-based working solutions, these concentrations are closer to those found in human plasma, the portal circulation, and the intracellular fluid of enterocytes following fruit or vegetable consumption [48,49]. Thus, the use of aqueous solutions of potential antioxidants better reflects the in vivo conditions in human plasma.

## Figures and Tables

**Table 1:** Effect of selected organic solvents on UPE in the complete (Fe^2+^-EGTA-H_2_O_2_) and incomplete (Fe^2+^-EGTA-H_2_O_2_) modified Fenton systems.

Solvent	Final Solvent Concentration in Reaction Milieu [µmol/L]	Complete System Fe^2+^-EGTA-H_2_O_2_		Incomplete System Fe^2+^-EGTA-H_2_O	
		Without Solvent	With Solvent	Without Solvent	With Solvent
**Solvents that inhibit light emission from Fe^2+^-EGTA-H_2_O_2_ system**					
Methanol	0.105	4217 ± 79 (4203; 109) *	1609 ± 130 (1563; 109)	753 ± 18 (756; 21)	786 ± 82 (756; 21)
	0.21	3925 ± 164 (3871; 108) *	1202 ± 316 (1355; 365)	740 ± 32 (727; 46)	734 ± 17 (730; 23)
	0.42	3109 ± 340 (3237; 353) *	1142 ± 65 (1171; 60)	654 ± 21 (655; 15)	626 ± 11 (630; 16)
Ethanol	0.105	3971 ± 248 (3937; 198) *	1890 ± 35 (1879; 23)	812 ± 26 (815; 38)	789 ± 15 (787; 19)
	0.21	4062 ± 91 (4033; 114) *	1735 ± 56 (1746; 52)	733 ± 20 (723; 16)	725 ± 12 (728; 15)
	0.42	3765 ± 165 (3778; 262) *	1331 ± 47 (1337; 54)	642 ± 21 (633; 31)	618 ± 23 (627; 27)
Propan-1-ol	0.105	4214 ± 78 (4203; 101) *	1457 ± 120 (1447; 90)	672 ± 25 (673; 27)	660 ± 16 (651; 23)
	0.21	4136 ± 175 (4254; 244) *	1369 ± 86 (1392; 91)	707 ± 16 (712; 27)	710 ± 18 (715; 24)
	0.42	3407 ± 198 (3389; 119) *	1222 ± 58 (1230; 72)	617 ± 14 (615; 10)	640 ± 62 (631; 46)
n-Pentanol	0.105	2999 ± 206 (2939; 103) *	1975 ± 106 (1967; 129)	761 ± 31 (754; 20)	752 ± 52 (737; 18)
	0.21	2829 ± 164 (2884; 172) *	2116 ± 139 (2084; 1510)	690 ± 15 (684; 15)	679 ± 13 (680; 17)
	0.42	2801 ± 159 (2741; 137) *	1825 ± 55 (1819; 25)	607 ± 17 (595; 30)	579 ± 39 (567; 22)
Acetonitrile	0.105	3243 ± 361 (3353; 164)	3249 ± 52 (3266; 49)	794 ± 14 (787; 18)	778 ± 11 (780; 18)
	0.21	3550 ± 110 (3577; 173) *	2894 ± 51 (2872; 62)	699 ± 20 (704; 30)	705 ± 52 (683; 88)
	0.42	3347 ± 188 (3254; 1420) *	2576 ± 65 (2560; 92)	603 ± 20 (601; 28)	572 ± 13 (572; 16)
DMSO	0.105	3624 ± 242 (3650; 170) *	1207 ± 46 (1197; 34)	759 ± 52 (738; 26)	754 ± 23 (761; 37)
	0.21	3921 ± 969 (3932; 663) *	1200 ± 54 (1198; 71)	708 ± 18 (705; 20)	690 ± 19 (694; 15)
	0.42	3641 ± 193 (3722; 172) *	1072 ± 36 (1057; 46)	625 ± 18 (631; 26)	587 ± 20 (588; 12)
Benzyl acetate	0.105	3224 ± 144 (3246; 106) *	2105 ± 131 (2116; 85)	580 ± 17 (578; 26)	551 ± 22 (560; 25)
	0.210	3125 ± 133 (3174; 171) *	2144 ± 27 (2143; 29)	689 ± 15 (681; 19)	674 ± 14 (681; 13)
	0.42	3173 ± 107 (3205; 124) *	2017 ± 65 (2019; 87)	580 ± 17 (578; 26)	551 ± 22 (560; 25)
**Solvents that enhance light emission from Fe^2+^-EGTA-H_2_O_2_ system**					
Acetone	0.105	3270 ± 96 (3243; 103) *	4427 ± 135 (4454; 71)	704 ± 17 (703; 20)	715 ± 29 (711; 42)
	0.21	3015 ± 176 (2950; 197) *	4174 ± 96 (4165; 110)	681 ± 15 (682; 10)	672 ± 19 (669; 20)
	0.42	3240 ± 221 (3299; 271) *	4179 ± 99 (4194; 96)	639 ± 14 (637; 11)	617 ± 14 (615; 21)
Ethyl acetate	0.105	3260 ± 184 (3196; 155) *	5960 ± 196 (5932; 167)	467 ± 20 (461; 33)	463 ± 13 (459; 22)
	0.21	3528 ± 149 (3492; 148) *	5377 ± 141 (5303; 193)	710 ± 10 (714; 11)	720 ± 5 (721; 5)
	0.42	3043 ± 360 (3209; 500) *	5280 ± 238 (5176; 3330	731 ± 18 (733; 27)	702 ± 11 (705; 18)
Amyl acetate	0.105	3478 ± 222 (3439; 207) *	4623 ± 213 (4651; 160)	747 ± 18 (752; 24)	749 ± 14 (750; 14)
	0.21	3057 ± 159 (3082; 135) *	4153 ± 261 (4063; 380)	736 ± 25 (731; 27)	739 ± 18 (733; 26)
	0.42	3125 ± 91 (3121; 66) *	4193 ± 187 (4215; 98)	595 ± 29 (600; 410	570 ± 18 (562; 19)
Butyl butyrate	0.105	3364 ± 117 (3406; 167) *	6475 ± 462 (6372; 680)	747 ± 18 (752; 24)	749 ± 14 (750; 14)
	0.21	3315 ± 110 (3307; 163) *	18,124 ± 1412 (18,633; 1751)	727 ± 14 (727; 17)	739 ± 28 (730; 17)
	0.42	3303 ± 137 (3295; 204) *	19,338 ± 738 (19,310; 761)	686 ± 10 (687; 11)	700 ± 21 (705; 20)

**Table 2:** Inhibitory effect of selected organic solvents at final concentrations of 0.105 µmol/L, 0.21 µmol/L, and 0.42 µmol/L on UPE in 92.6 µmol/L Fe^2+^-185.2 µmol/L EGTA-2.6 mmol/L H_2_O_2_ system.

Solvent	Chemical Structure	% Inhibition			Graph
		0.105 µmol/L	0.21 µmol/L	0.42 µmol/L	
Methanol	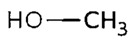	62 ± 4 * (62; 2)	69 ± 8 * (65; 9)	63 ± 4 * (64; 3)	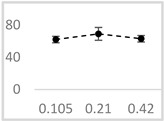
Ethanol	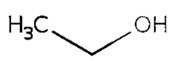	52 ± 3 * (52; 3)	57 ± 2 * (57; 20	65 ± 1 * (64; 2)	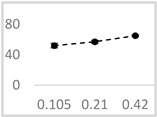
Propan-1-ol	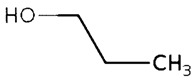	65 ± 2 * (66; 2)	67 ± 2 * (66; 3)	64 ± 3 * (64; 4)	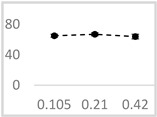
n-Pentanol	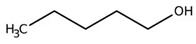	34 ± 6 * (32; 0)	25 ± 5 * (26; 0)	35 ± 2 * (32; 0)	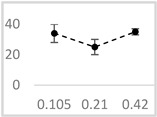
Acetonitrile	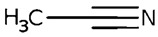	−1 ± 13 (2; 0)	18 ± 3 * (19; 0)	23 ± 5 * (21; 0)	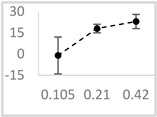
DMSO	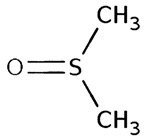	67 ± 2 * (67; 1)	68 ± 7 * (67; 5)	70 ± 2 * (71; 3)	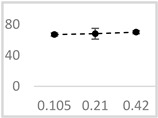
Benzyl acetate	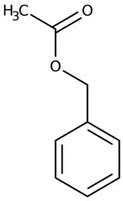	35 ± 8 * (35; 6)	31 ± 3 * (31; 4)	36 ± 4 * (37; 2)	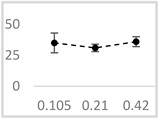

UPE—ultra-weak photon emission expressed in Relative Light Units (RLU). The tested solvent was mixed with EGTA and Fe^2+^, followed by the automatic injection of H_2_O_2_. The total light emission was then measured for 120 s. The results are presented as the mean and standard deviation (median; interquartile range) of the percentage inhibition of light emission, which was obtained from seven independent experiments. *—significant inhibition (*p* < 0.05) compared to the UPE of the Fe^2+^-EGTA-H_2_O_2_ system without the solvent.

**Table 3:** Enhancing effect of selected solvents at final concentrations of 0.105 µmol/L, 0.21 µmol/L, and 0.42 µmol/L on UPE in 92.6 µmol/L Fe^2+^-185.2 µmol/L EGTA-2.6 mmol/L H_2_O_2_ system.

Solvent	Chemical Structure	% Enhancement of UPE			Graph
		0.105 µmol/L	0.21 µmol/L	0.42 µmol/L	
Acetone	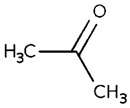	36 ± 7 * (39; 8)	39 ± 6 * (40; 5)	30 ± 12 * (25; 14)	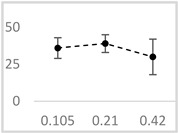
Ethyl acetate	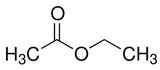	83 ± 11 * (84; 12)	53 ± 8 * (52; 12)	75 ± 20 * (75; 24)	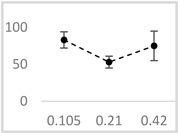
Amyl acetate	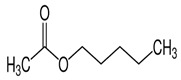	33 ± 8 * (35; 8)	36 ± 10 * (40; 15)	34 ± 8 * (37; 10)	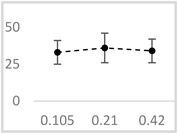
Butyl butyrate	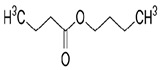	93 ± 16 * (93; 12)	446 ± 35 * (452; 40)	487 ± 40 * (496; 53)	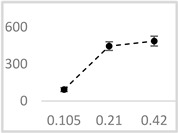

UPE—ultra-weak photon emission expressed in Relative Light Units (RLU).

**Table 4:** Design of experiments on the effect of various organic solvents on light emission in the Fe^2^⁺-EGTA-H_2_O_2_ system.

Number	Sample	Working Solutions Added to Luminometer Tube (µL)					
		A PB	B EGTA	C FeSO_4_	D Solvent	E H_2_O_2_	F H_2_O
1	Complete system	940	20	20	0	100	0
2	Complete system + solvent	920	20	20	20	100	0
3	Incomplete system	940	20	20	0	0	100
4	Incomplete system + solvent	920	20	20	20	0	100

## Data Availability

The data supporting the findings of this study are available upon request from the corresponding author.
